# Characterization of Substrate Preference for Slc1p and Cst26p in *Saccharomyces cerevisiae* Using Lipidomic Approaches and an LPAAT Activity Assay

**DOI:** 10.1371/journal.pone.0011956

**Published:** 2010-08-04

**Authors:** Guanghou Shui, Xue Li Guan, Pradeep Gopalakrishnan, Yangkui Xue, Joyce Sze Yuin Goh, Hongyuan Yang, Markus R. Wenk

**Affiliations:** 1 Department of Biochemistry, National University of Singapore, Singapore, Singapore; 2 Life Science Institute, National University of Singapore, Singapore, Singapore; 3 Department of Biological Sciences, National University of Singapore, Singapore, Singapore; 4 School of Biotechnology and Biomolecular Sciences, University of New South Wales, Sydney, New South Wales, Australia; Cinvestav, Mexico

## Abstract

**Background:**

Phosphatidic acid (PA) is a key regulated intermediate and precursor for *de novo* biosynthesis of all glycerophospholipids. PA can be synthesized through the acylation of lysophosphatidic acid (LPA) by 1-acyl-3-phosphate acyltransferase (also called lysophosphatidic acid acyltransferase, LPAAT). Recent findings have substantiated the essential roles of acyltransferases in various biological functions.

**Methodologies/Principal Findings:**

We used a flow-injection-based lipidomic approach with ∼200 multiple reaction monitoring (MRM) transitions to pre-screen fatty acyl composition of phospholipids in the yeast *Saccharomyces cerevisiae* mutants. Dramatic changes were observed in fatty acyl composition in some yeast mutants including Slc1p, a well-characterized LPAAT, and Cst26p, a recently characterized phosphatidylinositol stearoyl incorporating 1 protein and putative LPAAT in *S. cerevisiae*. A comprehensive high-performance liquid chromatography–based multi-stage MRM approach (more than 500 MRM transitions) was developed and further applied to quantify individual phospholipids in both strains to confirm these changes. Our data suggest potential fatty acyl substrates as well as fatty acyls that compensate for defects in both Cst26p and Slc1p mutants. These results were consistent with those from a non-radioactive LPAAT enzymatic assay using C17-LPA and acyl-CoA donors as substrates.

**Conclusions:**

We found that Slc1p utilized fatty acid (FA) 18:1 and FA 14:0 as substrates to synthesize corresponding PAs; moreover, it was probably the only acyltransferase responsible for acylation of saturated short-chain fatty acyls (12:0 and 10:0) in *S. cerevisiae*. We also identified FA 18:0, FA 16:0, FA 14:0 and exogenous FA 17:0 as preferred substrates for Cst26p because transformation with a GFP-tagged *CST26* restored the phospholipid profile of a *CST26* mutant. Our current findings expand the enzymes and existing scope of acyl-CoA donors for glycerophospholipid biosynthesis.

## Introduction

Glycerophospholipids are major components of cellular membranes and are involved in a wide range of biological processes[1]. Most naturally occurring glycerophospholipids have a heterogeneous intramolecular acyl chain composition, usually with saturated acyls at the sn-1 position and unsaturated acyls at *sn*-2. Acyltransferases including glycerol-3-phosphate acyltransferase (GPAT), 1-acyl-3-phosphate acyltransferase (AGPAT) (also called lysophosphatidic acid acyltransferase, LPAAT), LPLAT (lysophospholipid acyltransferase) and diacylglyceride acyltransferase families play essential roles in acyl substrate selection for deacylation and reacylation reactions (Lands cycle) during glycerophospholipid biosynthesis [2]. Two pathways for *de novo* glycerophospholipid biosynthesis have been reported, using either glycerol-3-phosphate or dihydroxyacetone phosphate and acyl-CoA as substrates to synthesize lysophosphatidic acids (LPAs), the initial step of phospholipid biosynthesis [3]. Lysophosphatidic acid is further esterified at the sn-2 position with an acyl group to form phosphatidic acid (PA), which is catalyzed by LPAAT. Recent findings have further substantiated the essential roles of acyltransferases in membrane diversity, curvature, and asymmetric distribution of fatty acyls (FA) within individual phospholipids[4]–[6]. In particular, LPAAT plays important roles in various organisms[7]–[14]. Mutations in *AGPAT2* (or *LPAAT-β*) cause congenital generalized lipodystrophy, a syndrome with complete or nearly complete loss of adipose tissue evident at birth [8]. The affected children are prone to develop diabetes, hepatic steatosis, and hyperlipidemia with age [10]. A recent study of Agpat2(−/−) mice suggested that both dietary fat and hepatic triglyceride (TAG) biosynthesis via a monoacylglycerol pathway may contribute to hepatic steatosis in these mice [9]. In contrast, increased expression of LPAAT-*β* (AGPAT2) is associated with malignancy[11]. As such, LPAAT-*β* may be an important enzyme in human tumor proliferation and tumor survival[12]. LPAAT-*β* expression is emerging as a prognostic marker and therapeutic target in gynecologic malignancies [7]. *AGPAT6* deficiency in mice reduces body weight and white adipocyte size, alters fatty acid composition, and causes subdermal lipodystrophy and resistance to obesity [13]. Plastid LPAAT is essential for embryo development in Arabidopsis during the transition from the globular to the heart stage, and loss of plastid LPAAT causes embryo lethality[14].

In *Saccharomyces cerevisiae*, PA can be synthesized *de novo* through the acylation of glycerol-3-phosphate by Gat1p (also known as Gpt2p) or Gat2p (also known as Sct1p) and subsequent acylation of LPA by Slc1p or Slc4p (also known as Ale1p, Lpt1p, Lca1p) [5]; [15]; [16]. A few recent studies have revealed that the *S. cerevisiae* gene *SLC4* or *LPT1* encodes an acyl-CoA-dependent LPLAT capable of acylating LPA, lysophosphatidylcholine, lysophosphatidylethanolamine, lysophosphatidylglycerol, lysophosphatidylinositol and lysophosphatidylserine [17]. *SLC1*, together with *SLC4*, encodes partially redundant LPAAT activity in *S. cerevisiae* [16]. The *slc1Δslc4Δ* double knockout is lethal, indicating that LPAAT activity is essential in yeast [16].

Current approaches for evaluating acyltransferase activity are mainly based on enzymatic assays monitored by quantification of radioactive compounds via thin-layer chromatography [15]–[18]. In recent years, mass spectrometry (MS)–based lipidomics approaches have been commonly used to study lipid metabolism in various biological systems. For instance, shotgun lipidomic approaches are widely used to study lipid metabolism in various models, mainly based on unbiased profiling and tandem MS for individual classes of lipids [19]–[21]. In this study, we used FA-based multiple reaction monitoring (MRM) approaches to screen for potential acyltransferases in the EUROpean Saccharomyces Cerevisiae ARchive for Functional analysis (EUROSCARF) library to characterize potential substrates for annotated acyltransferase genes. We found dramatic changes in fatty acyl profile in some mutants, such as Δ*SLC1* and Δ*CST26*, compared with wild-type yeast. Data from our lipidomic analysis will provide information on the substrate (acyl chain) specificity of any acyltransferase. Further, we correlated our lipidomics results with data from acyltransferase activity assays.

## Results

**Figure 1:**
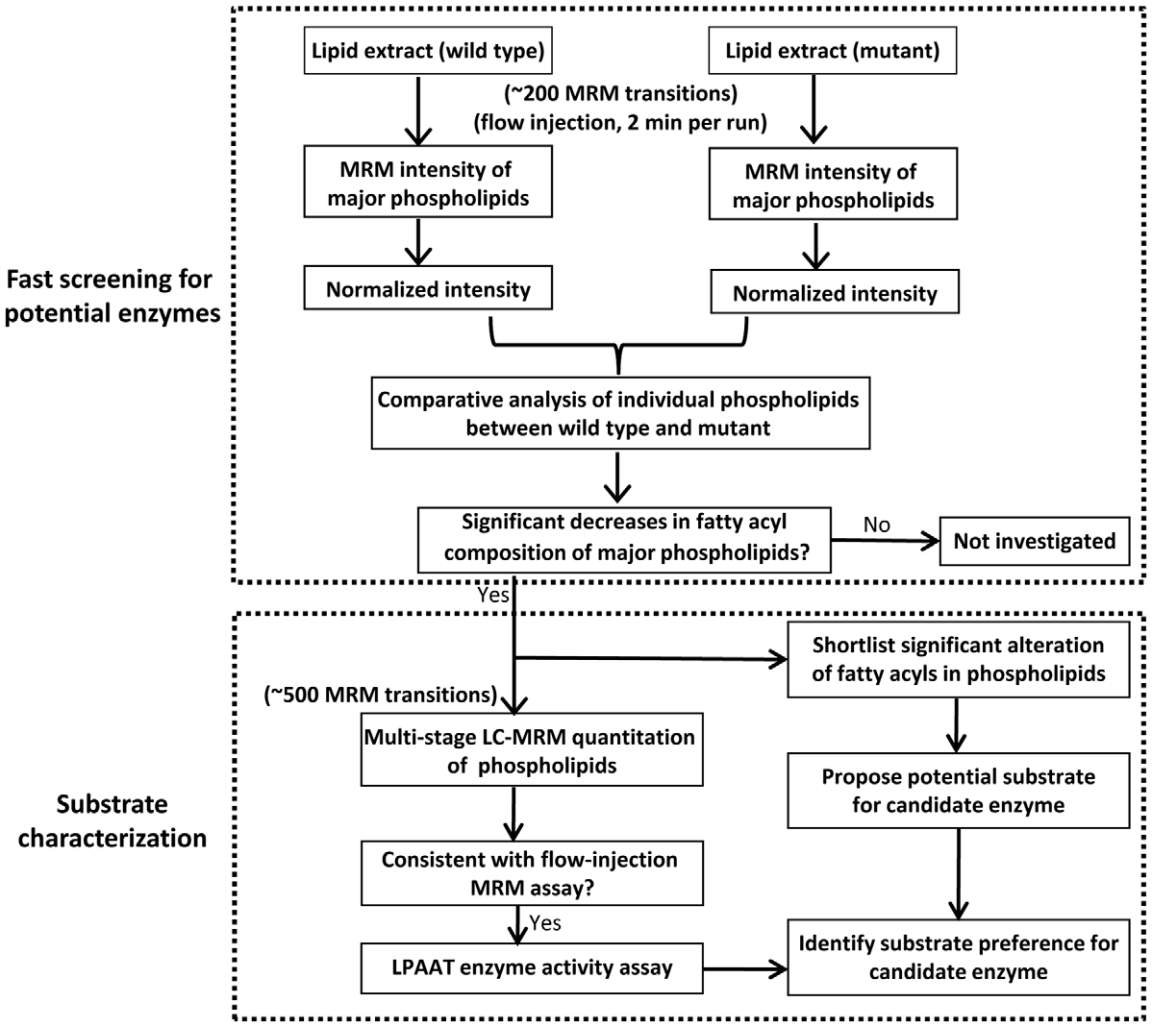
Flow chart illustrating the lipidomic approach to screen and characterize fatty acyl substrates for potential acyltransferases.

**Figure 2:**
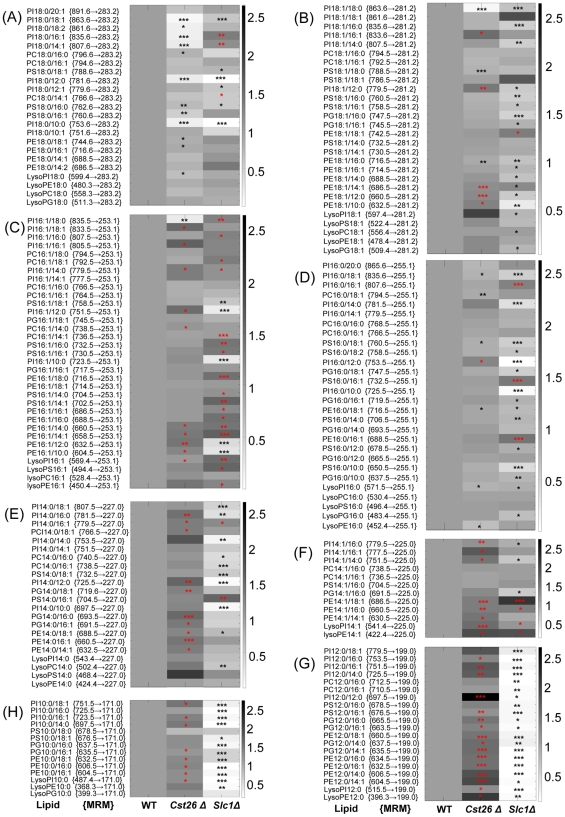
Fatty acyl–based MRM analysis of individual lipids in *cst26*Δ and *slc1*Δ. Heat plot shows differential levels of individual lipid species in mutants, as indicated by MRM measurement, compared with wild-type cells. (A) FA-18:0-containing phospholipids; (B) FA-18:1-containing phospholipids; (C) FA-16:1-containing phospholipids; (D) FA-16:0-containing phospholipids; (E) FA-14:0-containing phospholipids; (F) FA-14:1-containing phospholipids; (G) FA-12:0-containing phospholipids; (H) FA-10:0-containing phospholipids. *, p<0.05; **, p<0.005; ***, p<0.001 (significant increases are indicated by red asterisks, and significant decreases by black asterisks).

**Figure 3:**
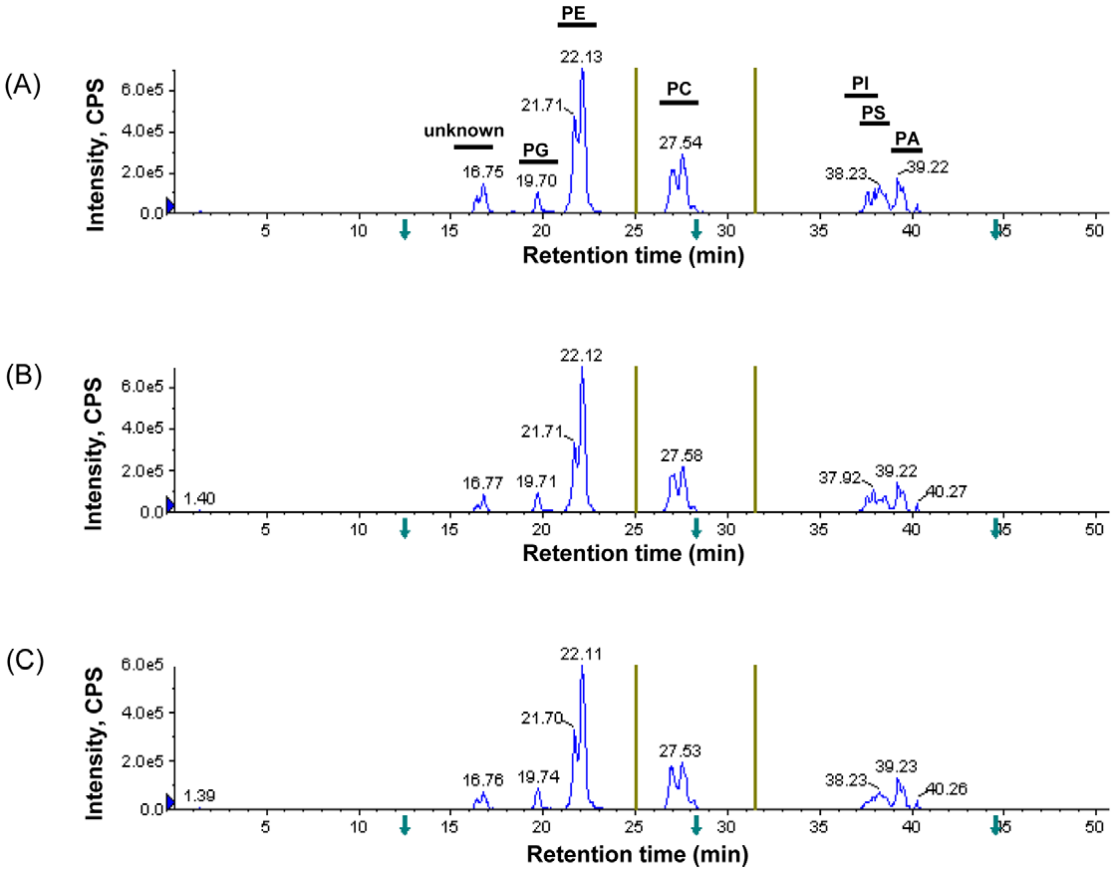
Multi-stage MRM analysis of individual lipids in each class (listed in Table S1) after normal-phase LC separation. (A) wild type; (B) *ΔCST26*; (C) *ΔSLC1*. PG, phosphatidylglycerol; PE, phosphatidylethanolamine; PC, phosphatidylcholine; PI, phosphatidylinositol; PS, phosphatidylserine; PA, phosphatidic acid. Y-axis: intensity, counts per second (CPS). The Olive lines on the left and right side of PC show switching time points for scheduled MRM transitions.

We used an integrated lipidomic-based approach to directly characterize potential fatty acyl substrates for known or putative LPAAT acyltransferases (Figure 1). Briefly, flow-injection-based MRM analysis of individual lipid species was utilized for rapid screening of altered fatty acyls in the yeast *S. cerevisiae* mutants (Figure 2). Selected strains, which in prescreening showed significantly altered fatty acyl profiles in phospholipids, were further investigated using a multi-stage LC-MRM approach (over 500 MRM transitions) for analysis of individual lipids to confirm the results obtained through rapid-flow injection (Figure 3). Lastly, LPAAT enzymatic activity was measured to confirm results obtained by lipidomic analyses.

### Sensitive fatty acyl–based MRM analysis of crude lipid extract from yeast

Previous studies on phospholipid species in the yeast *S. cerevisiae* indicated that it has a relatively simple phospholipid composition[21]–[23]. Most MRM-based lipidomic approaches have been based on the MRM transition to polar head groups, which provides information on both of the polar head groups and total number of carbon-carbon double bonds of the two fatty acyl chains in a phospholipid. Despite the relatively high sensitivity of this approach, it does not yield detailed information on each fatty acid. An additional screen of fatty acyl–based precursor ion scan is usually required to obtain such information.

We used fatty acyl–based MRM transitions to screen for individual phospholipids in yeast. The MRM transitions were classified into different groups based on yeast fatty acyl composition, i.e., FA 18:0, 18:1, 16:0, 16:1, 14:0, 14:1, 12:0 and 10:0. In the negative ESI mode, PE species ionize more readily than PC species. With piperidine as a modifier, ionization of PE species was enhanced by ∼10 fold, which was much greater than increases in ionization of PC species. Thus, it may be possible to qualitatively profile PE species directly using MRM transitions of [M−H]^−1^ over individual fatty acyl ions[20]. PC could be detected using [M+Cl]^−1^ without overlapping with other phospholipids. Thus, we applied chloroform:methanol:200 mM piperidine (1∶1∶0.05) as a mobile phase to directly introduce the sample into the mass spectrometer for MRM analysis. This approach is advantageous for the following reasons: (1) enhanced ionization of all phospholipids, in particular PE and PS species in the negative ESI mode [20]; (2) fatty acyl–based MRM yields better sensitivity than polar-head MRM transitions; and (3) selection of an appropriate mobile phase dramatically reduces carryover of sticky phosphatidylserine in the machine lines and generates better peak shape (data not shown), which facilitates high-throughput screening. Furthermore, using an autosampler to manipulate samples prevents manual error and is thus labor efficient.

### Comprehensive analysis of individual lipids using a multi-stage LC-MRM approach

Although the flow-injection-based MRM approach serves as a rapid way to prescreen for the presence of altered fatty acyls in yeast mutants, more accurate quantitation of certain lipid species, in particular PE and PC, requires well-resolved chromatography separation. We used a Luna silica column and a typical normal-phase mobile phase system to develop a multi-stage LC-MRM approach with over 500 MRM transitions in a 50-min run and demonstrated that different classes of phospholipids could be successfully separated. The rather large linear range using MRM for quantitation allowed the accurate analysis of major yeast phospholipids in a single run. Among those phospholipids validated, PG eluted first, followed by PE, PC, PI, PS and PA. PI and PS were not completely resolved using this elution system, but the different MRM transitions for these two classes of lipids in yeast allowed their quantitation in a single run (Figure 3 and Table S1). Total ion chromatograms of *cst26Δ* and *slc1Δ* cells were similar to that of wild-type (Figure 3A–C). Levels of individual lipid species were calculated by comparison with spiked internal standards.

### Characterization of potential substrates for Slc1p using mass spectrometry

Slc1p is the primary 1-acyl-sn-glycerol-3-phosphate acyltransferase mediating the incorporation of unsaturated acyl chains into phospholipids during *de novo* synthesis [16]. As a positive control, we examined whether a lipidomics approach could directly detect Slc1p function, with confirmation by an *in vitro* radioactive enzymatic assay.

ESI-MS was first applied to profile lipids in crude extracts of wild-type and *slc1Δ* mutant of *S. cerevisiae*, and the results (Figure S1A–C) were similar to our previous findings [24]. The *slc1Δ* cells showed dramatic decreases in PI 30:0 (m/z 781), PI 28:0 (m/z 753), PI 28:0 (m/z 725), LysoPI 14:0 (m/z 545) and LysoPI 12:0 (m/z 517). Further precursor ion scanning based on the fragment of phosphatidylinositol (m/z 241.0, C_6_H_10_O_8_P) for PI profiles revealed the absence of saturated short-chain PI species including PI 26:0, 28:0, 30:0, etc (Figure S1D–E). We next applied the fatty acyl–based MRM approach to detect alterations in phospholipid fatty acyl chains in crude lipid extracts of *slc1Δ* cells. Not surprisingly, the composition of fatty acyls in *slc1Δ* phospholipids differed substantially from that of wild type (Figure 2A–H). Of the changes among the major fatty acyls, the levels of most 18∶1-contaning phospholipids were significantly lower in *slc1Δ*, whereas the levels of most 16∶1-containing phospholipids were significantly higher (Figure 2B, 2C). The levels of most phospholipids containing FA14:0 were significantly decreased (Figure 2D, 2E). In addition, phospholipids containing short-chain fatty acyls, such as FA 12:0 and FA 10:0, were nearly absent in *slc1Δ* (Figure 2G, 2H). By contrast, most phospholipids containing FA 16:1 and FA 14:1 were elevated in *slc1Δ* (Figure 2C, 2F). Similar profile changes were observed using normal-phase LC in combination with the multi-stage MRM approach (Figure 3 and data not shown).

### Characterization of potential substrates for Cst26p using MS

Cst26p contains a 1-acylglycerol-3-phosphate acyltransferase domain (Figure S2) and thus is a putative acyltransferase. We investigated whether Cst26p had acyltransferase activity in the yeast *S. cerevisiae* and, if so, which fatty acyls were its potential substrates.

ESI-MS was first applied to profile lipids in crude extracts of wild-type and *cst26Δ* of *S. cerevisiae*. Comparison of the normalized lipid profiles for the wild type and *cst26Δ* revealed a dramatic reduction in lipid species with m/z at 863 (PI 36:1) (Figure S3A–B, representative data from rich medium). A differential log ratio plot was generated to further compare wild-type and *cst26*Δ lipid profiles, and ions at m/z 863 (PI 36:1) were the most substantially regulated peaks (Figure S3C). There were a few other differences as well, such as decreases in PI 34:1, lysoPI 18:0 and lysoPI 16:0 in the mutant, and increases in PE 32:2, PI 32:2, PI 34:2 and lysoPI 16:1. These data indicate that deletion of *CST26* alters the fatty acyl composition of phospholipids. Further fatty acyl–based precursor ion scanning at m/z 283.2 showed a dramatic decrease in FA 18:0-containing phosphoinositides (Figure S3D–E).

The above-mentioned fatty acyl–based MRM approach was applied to qualitatively assess differences in the composition of certain individual fatty acyls in various phospholipids in *cst26Δ* cells. This approach allowed direct visualization of fatty acyl distribution in crude lipid extracts of yeast cells (Figure 2A–G, representative data from YPD medium). Distinct changes in fatty acyl composition among certain phospholipids were observed in the *CST26* mutant compared to wild type (Figure 2A–G), such as a substantial reduction in FA18:0-containing PI species and significant decreases in most other phospholipids containing a single FA18:0 (Figure 2A).

We did not observe dramatic changes in most phospholipid species containing FA18:1, except that PI18:0/18:1, PE18:1/16:0 and PS18:0/18:1 were decreased and a few short-chain FA-containing species were significantly increased (Figure 2B). However, no significant change was observed for all FA18:1-based lysophospholipids. Overall results from the FA 18:1-based MRM assay indicated that deletion of *CST26* does not alter the levels of FA 18:1 in cellular phospholipids (Figure 2B).

Significant decreases in most FA16:0-containing phospholipids and lysophospholipids were observed in the *CST26* mutant (Figure 2D), indicating that FA 16:0 is also a potential fatty acyl substrate of Cst26p. In contrast to the levels of FA 18:0- and FA 16:0-containing phospholipids in the *CST*26 mutant, most FA 16:1-containing phospholipids were elevated in the mutant compared with wild type (Figure 2C). Similar results were observed for the short-chain fatty acyl species 14:1, 12:0 and 10:0 (Figure 2E–G) and for cells cultured in SC medium. Further normal-phase LC in combination with multi-stage MRM analysis showed similar trends to those observed in the flow-injection MRM analysis (data not shown).

### Cst26p localizes to lipid droplets, and *cst26Δ* cells expressing GFP-tagged *CST26* have a normal lipid profile

**Figure 4:**
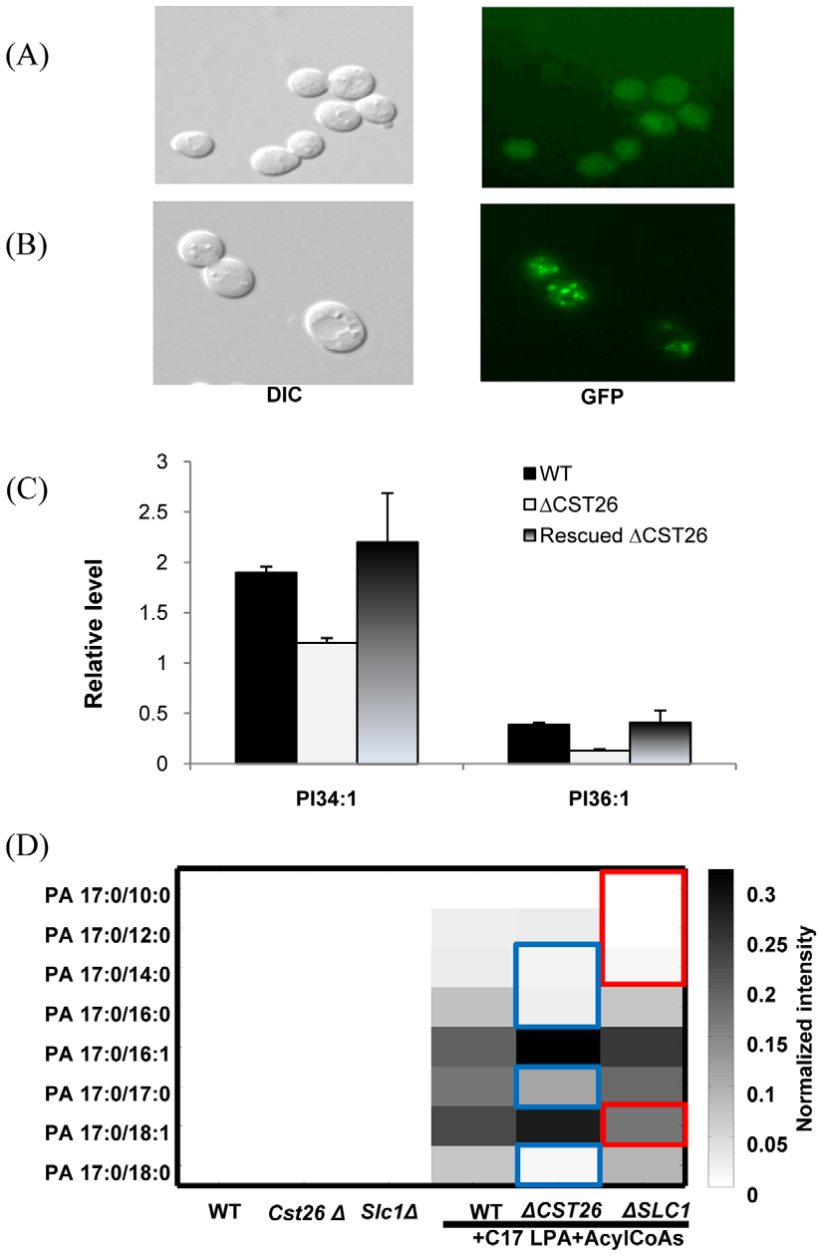
Localization of Cst26p in *Δcst26* cells transformed with GFP-tagged Cst26 and LPAAT activity assay. (A) Micrographs of *ΔCST26* cells transformed with the empty vector. DIC, differential interference contrast. (B) flurescence microscopy of*ΔCST26* cells transformed with GFP-tagged Cst26. (C) PI 34:1 and PI36:1 levels were restored in the *CST26*-transformed *ΔCST26* mutant; (D) In the LPAAT activity assay, *CST26* shows acyltransferase activity toward 18:0, 17:0, 16:0, 14:0 (blue rectangles) with 16.7% of PA17:0/18:0, 65.1% of PA17:0/17:0, 28.5% of PA17:0/16:0 and 65.8% of PA 17:0/14:0 when compared with corresponding PA in wild type, whereas *SLC1* shows activity toward 10:0, 12:0, 14:0 and 18:1 (red rectangles) with 29% of PA17:0/10:0, 19% of PA17:0/12:0, 52% of PA17:0/14:0 and 76% of PA17:0/18:1 when compared with corresponding PA in wild type. Levels of individual PA species were expressed as normalized intensity to internal standard PA14:0/14:0.

To conclusively show that the observed changes in the lipid profile were due to deletion of *CST26*, we transformed a GFP-tagged Cst26p into a *CST26* mutant strain. Cst26p localized to lipid droplets (Figure 4A–B), as observed in GFP-tagged *CST26* transformed cells. Moreover, the lipid composition of the transformed cells was restored to that of wild-type cells, indicating that the GFP-tagged Cst26p compensated for the loss of function of the enzyme in the mutant. (Figure 4C, Figure S4).

### Characterization of the substrate for Slc1p and Cst26p as assessed by LPAAT activity

To further characterize the substrates of the two acyltransferases, we assessed LPAAT activity in the presence of a synthetic substrate, C17 LPA, and various other acyl-CoA substrates. Compared to wild type, *cst26Δ* cells produced significantly lower levels of PA17:0/18:0, PA17:0/16:0, and PA17:0/14:0, whereas *slc1Δ* cells had a defect in conversion of LPA into PA17:0/18:1, PA17:0/10:0, PA17:0/12:0 and PA17:0/14:0 (Figure 4D). Thus, the fatty acyl substrates for Cst26p include 18:0-acyl-CoA, 16:0-acyl-CoA, 17:0-acyl-CoA and 14:0-acyl-CoA, whereas those for Slc1p include 18:1-acyl-CoA, 14:0-acyl-CoA, 12:0-acyl-CoA and 10:0-acyl-CoA (Figure 4D).

### LC-MS analysis of neutral lipids in *cst26Δ* and *slc1Δ* cells

**Figure 5:**
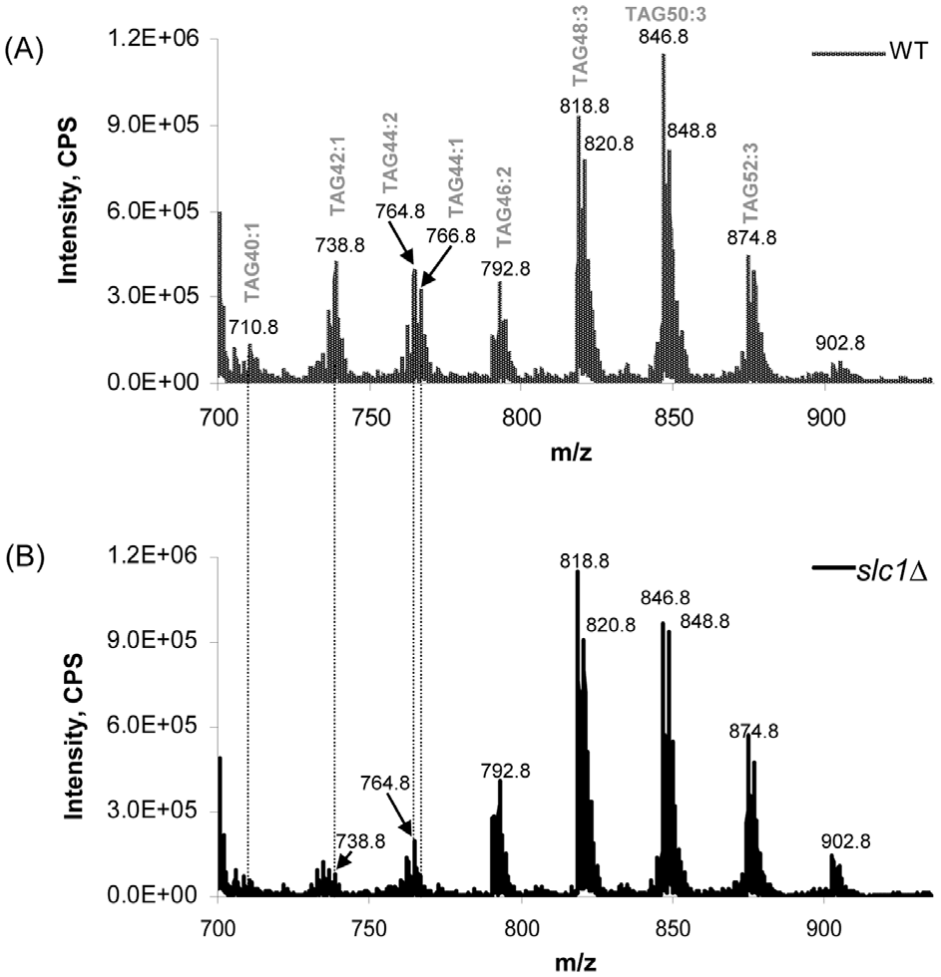
Triacylglycerol (TAG) profiles in *slc1Δ* cells. (A) wild-type; (B) *slc1Δ*. Levels of short-chain TAG species, including TAG40:1, TAG42:1 and TAG44:1, were dramatically decreased in the slc1-deficient mutant.

Dramatic decreases in short-chain TAG species, including TAG40:1, 42:1 and 44:1, were observed in *slc1Δ* cells, accompanied with an obvious increase in TAG48:3 (Figure 5). Short-chain diacylglycerol (DAG) species, including DAG26:0, 28:0 and 30:0, were also dramatically decreased (data not shown). Overall, changes in fatty acyl composition of both TAG and DAG species showed trends similar to those observed for phospholipids in *slc1Δ* cells. There were no significant changes in individual TAG or DAG species in *cst26Δ* cells compared with wild type (data not shown).

## Discussion

In recent years, MS has been widely used in a number of studies investigating lipid metabolism] [19]; [21]; [22]; [25]–[27]. In particular, the introduction of tandem MS has spurred the development of various quantitative approaches, such as precursor ion scanning, neutral loss scanning, and MRM scanning, for targeted lipidomic analysis of complex samples[22]; [28]; [29]. Precursor ion scanning of individual fatty acyls has been used to investigate fatty acyl profiles of phospholipids[30]. Fatty acyl composition of complex lipids can also be rapidly characterized by collision-induced dissociation MS [31], but this method does not provide information on fatty acyl composition among different classes of phospholipids and lysophospholipids. Recently, FA-based MRM approaches have been shown to provide such direct information[23]; [32]. In this study, we used chloroform:methanol (1∶1) containing 5 mM piperidine as a mobile phase and an autosampler to introduce crude lipid extract directly into a triple-quadrupole mass spectrometer, which allowed the sensitive and qualitative analyses of major yeast phospholipids within 2 min. This simple approach serves as a rapid tool to investigate potential fatty acyl substrates for known and putative/unknown acyltransferases in yeast. Of those potential acyltransferases, we screened (data not shown and ongoing work) fatty acyl profiles of *cst26p* and *slc1p*, which we found differed substantially from the wild-type profile (Figure 2A–H). Using this approach, we also observed dramatic changes in the profiles for other well-characterized acyltransferases, including *lpt1p* (data not shown and ongoing work).

However, PC species were poorly ionized in negative ESI mode, most likely due to ion suppression from other phospholipids (PI, PE and PS) when direct infusion or flow injection was utilized. A more comprehensive multi-stage LC-MRM approach was developed that provided in-depth and accurate analysis of individual lipid species in each class (Figure 3 and Table S1). Using a normal-phase column for lipid separation, the multi-stage LC-MRM increased MS signals of PC species by over 10 fold, allowing accurate quantification of most PC and PE species in a single LC-MS run. Provided with suitable internal standards, minor lipid species including lysophospholipid species could also be accurately quantified. For instance, lysoPE species were coeluted with PC species, while LysoPI, LsoPC, LysoPA and LysoPSspecies were eluted within the period from 40 to 50 min (data not shown).

Characterization of fatty acyl substrates for a potential acyltransferase using an enzymatic activity assay usually requires the purification of the protein or isolation of microsomal fractions as well as radioactive ^14^C-labeled glycerol 3-phosphate or ^14^C-labeled acyl-CoA [15]–[18]. Through direct analysis of cellular phospholipids using the LC-based fatty acyl MRM approach, we obtained additional and direct *in vivo* information that enabled rapid characterization of fatty acyl preference of a potential acyltransferase.

The decreased content of the major FA 18:1 in most phospholipids and lysophospholipids in *slc1Δ* cells suggests that FA 18:1 is a major substrate for Slc1p (Figure 2B). Increases in the content of other unsaturated fatty acyls such as FA 16:1 and FA 14:1 could be due to upregulation of another acyltransferase(s) that utilizes these unsaturated fatty acyls as substrates (Figure 2C–F). In addition, changes in fatty acyl composition of *slc1Δ* indicate that FA 14:0 is also potential substrate of this acyltransferase. Depletion of phospholipids containing short-chain fatty acyls suggests that Slc1p is the main acyltransferase for short-chain fatty acyl donors including FA 12:0 and FA 10:0 (Figure 2G, H). The results from our lipidomic analysis were consistent with the LPAAT enzymatic assay for Slc1p, which demonstrated that this acyltransferase incorporated FA 18:1, FA 16:0, FA 14:0, FA 12:0 and FA 10:0 into LPA (Figure 4D).

Recently, Guedard et al. reported that Psi1p (Cst26p) is responsible for the stearic acid enrichment that is characteristic of phosphatidylinositol in yeast, which explains why only PI species containing FA18:0 were dramatically decreased in yeast deleted for *CST26* [26]. However, a domain analysis of the acyltransferase encoded by *CST26* suggested that this enzyme is specifically involved in the conversion of LPA to PA (Figure S2). The specific changes in lipid composition in the *CST26* mutant indicated that the acyltransferase encoded by *CST26* is involved in the transfer of FA 18:0 and FA 16:0 (Figure 2A, D). Decreases in lysophospholipids 18:0 (lysoPI 18:0, lysoPE 18:0, lysoPC 18:0, and LysoPG 18:0) in *CST26* mutant cells indicated alternative pathways to sequester lysophospholipids, which would be expected to increase in cells lacking Cst26p. As a consequence of the decrease in FA 18:0 or FA 16:0 in cellular phospholipids, a relatively higher proportion of other FA was incorporated into phospholipids.

Fluorescence microscopy of GFP-tagged Cst26p indicated that the protein localized to lipid droplets (Figure 4A–B), consistent with previous results [33]. The levels of major species that were decreased in the *CST26* mutant (PI species with m/z 863 and 835) were restored in transformed cells, and the lipid profile of transformed cells was similar to that of wild type (Figure 4C, Figure S4).

**Figure 6:**
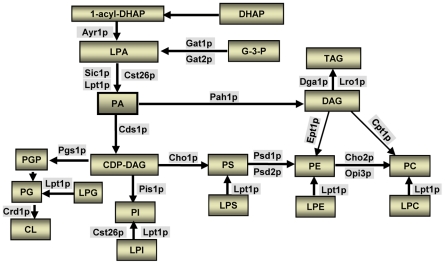
Biosynthesis of phospholipids in the yeast *S. cerevisiae*. G-3-P, glycerol 3-phosphate; DHAP, dihydroxyacetone phosphate; LPA, lysophosphatidic acid; PA, phosphatidic acid; DAG, diacylglycerol; TAG, triacylglycerol; PS, phosphatidylserine; LPS, lysophosphatidylserine; PE, phosphatidylethanolamine; LPE, lysophosphatidylethanolamine; PC, phosphatidylcholine; LPC, lysophosphatidylcholine; PI, phosphatidylinositol; LPI, lysophosphatidylinositol; PG, phosphatidylglycerol; PGP, phosphatidylglycerol phosphate; LPG, lysophosphatidylglycerol; CL, cardiolipin; CDP-DAG, cytidinediphosphate-diacylglycerol; Ayr1p, 1-acyl dihydroxyacetone phosphate reductase; Gat1p or Gat2p, Glycerol-3-phosphate acyltransferase; Slc1p, 1-acyl-sn-glycerol-3-phosphate acyltransferase (sphingolipid compensation); Lpt1p, lysophospholipid acyltransferase; Cst26, chromosome stability; Pah1p, Phosphatidic acid phosphohydrolase; Dga1p, Diacylglycerol acyltransferase; Lro1p, Lecithin cholesterol acyl transferase Related Open reading frame; Cds1p, CDP-diacylglycerol Synthase; Pgs1p, Phosphatidylglycerolphosphate synthase; Crd1p, Cardiolipin synthase; Pis1p, Phosphatidylinositol synthase; Cho1, Phosphatidylserine synthase (choline requiring); Psd1p/Psd2p, Phosphatidylserine decarboxylase; Cho2p, Phosphatidylethanolamine methyltransferase (choline requiring); Opi3p, Phospholipid methyltransferase (overproducer of inositol); Ept1p, sn-1,2-diacylglycerol ethanolamine- and cholinephosphotranferase; Cpt1p, Cholinephosphotransferase.

In the yeast *S. cerevisiae*, *SLC1* encodes the main LPAAT acyltransferase[5]. *SLC4* (*ALE1, YOR175C*), which encodes a membrane-bound *O*-acyltransferase and a lysophospholipid acyltransferase, is the key component of the Lands cycle for lysophospholipid acyltransferases (Figure 6) [16]; [17]; [34]; [35]. Interestingly, Slc1p has Mg^2+^-dependent acyltransferase activity toward lyso forms of phosphatidylserine and phosphatidylinositol, whereas *SLC4* also encodes a second LPAAT in *S. cerevisiae* [16]. Lipid analysis from *slc1Δ* and *slc4Δ* cells using traditional MS approaches revealed similar *in vivo* phospholipid profiles but did not convey information on fatty acyl composition [16]. Using our current approach, we systematically investigated the fatty acyl composition of phospholipids in the yeast *S. cerevisiae* and thus directly provided the probable *in vivo* function of *SLC1* and other genes. When applied to *cst26*Δ cells, our data indicated that Cst26p is another LPAAT that primarily uses FA 18:0 and FA 16:0 as the substrates in the yeast *S. cerevisiae*. These results were consistent with our LPAAT activity data, which showed that Cst26p is specific for saturated fatty acyl-CoAs (18:0, 17:0, 16:0, 14:0) whereas Slc1p is specific for FA 18:1, 14:0, 12:0, and 10:0 acyl-CoAs (Figure 4D). While both proteins possess an acyltransferase domain and prefer different fatty acyls as their main substrates, the lipid profile of the double knockout of SLC1 and YBR042C showed an additive effect that is all changes in SLC1 and YBR042C mutants (data not shown, ongoing project). There is no distinct phenotype change observed for both mutants. It will be interesting to look into effects of temperature shift as well as chemical stress on both mutants as well as double knockout mutant (ongoing project).

To the best of our knowledge, this is first evidence indicating that Cst26p is a LPAAT acyltransferase having FA18:0, 17:0, 16:0, and 14:0 acyl-CoA as its preferred substrates. We also report that Slc1p is likely the main acyltransferase to incorporate short-chain fatty acyls during the biosynthesis of phospholipids and TAGs in *S. cerevisiae*.

## Materials and Methods

### Chemicals

Chloroform and methanol were purchased from Merck (Merck Pte. Ltd., Singapore). Piperidine was purchased from Sigma-Aldrich (St. Louis, MO, USA). Deionized water was obtained from a MilliQ purification system (Millipore, Bedford, MA, USA).

### Yeast strains and media

The strains used for this study were purchased from the EUROSCARF library. BY4741 is a wild-type strain, whereas *Δslc1* and *Δcst26* are deletion mutants for genes encoding the respective acyltransferases. Strains were grown on YPD medium (1% yeast extract, 2% Bacto-peptone, and 2% glucose) or synthetic complete medium prepared as described [23]. The cells were grown to an OD (600 nm) of 0.8–0.9 and harvested by centrifugation at 3500 rpm for 2 min. Cell pellets were washed with MilliQ water and transferred to 2-ml Eppendorf tubes and stored at −80°C.

### *CST26* mutant transformation

The plasmid YCplac111-scGFP, with ampicillin and leucin selection markers, was used in the construction of a GFP tagged Cst26. The *CST26* gene with its native promoter was amplified with overhanging sequences containing restriction digestion sites for the enzymes BamH1 (5′GCGGGATCCAAAAATAAAACAATAAAGTT3′) and HindIII (5′GCGAAGCTTGCCCTCTTTGGATATGCAG3′). The PCR product and the plasmid Ycplac111 was ligated, and the resulting plasmid was isolated and transformed into the *CST26* mutant as described earlier[36].

### Fluorescence imaging of yeast cells

Fluorescence imaging was performed on a Leica DMLB microscope (Wetzlar, Germany) with a Curtis 100 fluorescent lamp. GFP signals were visualized with a 470/40-nm bandpass excitation filter, a 500-nm dichromatic mirror, and a 525/50-nm bandpass emission filter (Leica filter cube GFP).

### Lipid extraction

Lipids were extracted as described with a slight modification of a previously described method [37]. Briefly, 150 µl of acid-washed glass beads and 900 µl of chloroform∶methanol (1∶2) were added to the cell pellet and vortexed for 10 min at 4°C. The tubes were transferred into a vacuum container and incubated overnight at 4°C with shaking at 1100 rpm, then 300 µl of chloroform and 300 µl of H_2_O were added. The mixture was vortexed for 30 s and then incubated on ice for 2 min. The tubes were then centrifuged at 9000 rpm for 2 min, and the lower organic phase was collected. Chloroform (500 µl) and 50 µl of 2 M HCl were added for the second extraction. The two organic extracts were combined and dried using a Speed-Vac (Thermo Savant, Milford, USA). The dried lipid film was stored at −80°C and reconstituted in chloroform:methanol (1∶1) before analysis by high-performance liquid chromatography coupled with MS (HPLC/MS).

### Rapid prescreening of fatty acyl composition in yeast phospholipids using flow injection

An Agilent HPLC system coupled with an Applied Biosystems Triple Quadrupole/Ion Trap mass spectrometer (4000Qtrap, Foster City, CA) and Applied Biosystems Triple Quadrupole/Ion Trap mass spectrometer (3200Qtrap) were used to analyze individual lipids. Sample (20 µl) was directly introduced into the mass spectrometer by loop injections with chloroform:methanol:200 mM piperidine (1∶1∶0.05) as a mobile phase at a flow rate of 250 µl/min [20]; [23]. Mass spectrometry was recorded in the negative ESI modes, and ESI conditions were: turbo spray source voltage, −4500 V; source temperature, 250°C; GS1: 40.00, GS2: 30.00, curtain gas: 25. Based on product ion and precursor ion analysis of head groups and fatty acyls, a comprehensive list of MRM transitions was then generated to follow fatty acyl compositions of these lipids (parent → fatty acyl fragment transitions) (Figure 2) [23]. For phospholipids, phosphatidylinositol (PI), phosphatidylethanolamine (PE), phosphatidylserine (PS) and phosphatidylglycerol (PG), MRM transitions of [M−H]^−1^ over individual fatty acyl ions were directly used for profiling. For phosphatidylcholine, MRM transitions of [M+Cl]^−1^ over individual fatty acyl ions were used for PC comparison. The signal intensity of each MRM value was normalized to the sum of MRM intensities of all species. Analysis was carried out in quadruplicate for each genotype, using individual lipid extracts as per culture. The Student t-test was used to determine the statistical significance of differences between values.

### Quantitative analysis of lipids using HPLC/MS

Although the above-described approach provides a fast, sensitive means to screen for substrates of potential acyltransferases, individual lipid species were further analyzed after normal-phase LC separation. Individual classes of polar lipids were separated using an Agilent 1200 HPLC system and a 3200 Q-Trap mass spectrometer (Applied Biosystems). The HPLC system contained an Agilent 1200 binary pump, an Agilent 1200 thermo sampler, and an Agilent 1200 column oven (Shui et al., unpublished work). HPLC conditions: Luna 3-µm silica column (i.d. 150×2.0 mm); mobile phase A (chloroform:methanol:ammonium hydroxide, 89.5∶10∶0.5), B (chloroform:methanol:ammonium hydroxide:water, 55∶39∶0.5∶5.5); flow rate 300 µl/min; 5% B for 3 min, then linearly switched to 30% B in 24 min and maintained for 5 min, and then linearly changed to 70% B in 5 min and maintained for 7 min. Then, the composition of the mobile phase was returned to the original ratio] over 5 min and maintained for 6 min before the next sample was analyzed. Mass spectrometry conditions were similar to those described above for the 4000 Qtrap MS. Over 500 MRM transitions for individual PG, PE, PC, PI, PS and PA species were set up at different elution stages for LC-MS analysis (Table S1). Individual lipid species were quantified by comparison with spiked internal standards PC-19:0/19:0, PE-17:0-17:0, PS-14:0/14:0, PA-17:0/17:0, PG-14:0/14:0, which were obtained from Avanti Polar Lipids (Alabaster, AL, USA). Dioctanoyl phosphatidylinositol PI-8:0/8:0 (Echelon Biosciences, Inc., Salt Lake City, UT, USA) was used to quantify phosphatidylinositol.

Neutral lipids were analyzed by HPLC/ESI/MS [37]. Triglycerides were separated from polar lipids on an Agilent Zorbax Eclipse XDB-C18 column (i.d. 4.6×150 mm). Selective ion monitoring was used to record major phospholipids, sterols and TAG species.

### Assay of LPAAT activity for Slc1p and Cst26p

The acyltransferase assay was performed as described by Gijon et al (32) using microsomes from yeast with some modifications. Briefly, LPAAT activity was measured by the transfer of acyl-CoAs to LPA to form PA. Reactions contained 50 mM Tris-HCl (pH 7.5), 1 mM EDTA, 18:0, 18:1, 17:0, 16:1, 16:0, 14:0, 12:0, or 10:0 FA-CoAs (0.04 µM each), 25 µM of C17-LPA, and microsomes from yeast (30 µg of protein) in a total volume of 200 µl. After incubation at 37°C for 10 min, reactions were stopped by the addition of 300 µl of chloroform∶methanol (1∶2). Analysis of PA was performed using fatty acyl–based MRM analysis as described above and HPLC-based approaches with MRM of the following specific transitions: m/z 689.6→269.2, 687.6→269.2, 675.6→269.2, 661.6→269.2, 659.6→269.2, 633.6→269.2, 605.5→269.2 and 577.5→269.2 for PA17:0/18:0, PA17:0/18:1, PA17:0/17:0, PA17:0/16:0, PA17:0/16:1, PA17:0/14:0, PA17:0/12:0 and P17:0/10:0, respectively. FA-based MRM transitions for PA analysis were also set up using fragmental ions of incorporated fatty acyls 18:0 (m/z 283.2), 18:1 (m/z 281.2), 16:0 (m/z 255.2), 16:1 (m/z 253.2), 14:0 (m/z 227.2), 12:0 (199.1) and 10:0 (FA171.1), respectively. Levels of individual PA species were calculated using spiked internal standard PA14:0/14:0 (Avanti) with an MRM transition of 591.5→227.2.

## Supporting Information

Table S1 MRM transitions of individual phospholipids.
(0.08 MB PDF)

Figure S1 Unbiased lipid profiling of the SCL1 mutant. (A) Normalized lipid profile for the wild type; (B) normalized lipid profile for the SCL1 mutant; (C) differential plot of the SCL1 mutant compared to wild type; (D) and (e) precursor ion scan of ions at m/z 241 in wild type (WT) and ΔSLC1, respectively.
(0.59 MB PDF)

Figure S2 CST26 contains an acyltransferase domain. The domain information of YBR042C shows that it possesses a 1-acylglycerol-3-phosphate acyltransferase domain PTHR10983.
(0.05 MB PDF)

Figure S3 Unbiased lipid profiling of *CST26* mutant. (A) Normalized lipid profile for the wild type; (B) normalized lipid profile for *CST26* mutant; (C) differential plot of *ΔCST26* compared to wild type; precursor ion scan of ions at m/z 283.1 (stearic acid) in wild-type (D) and *cst26Δ* cells (E).
(0.31 MB PDF)

Figure S4 Mass spectrometric profile of a *CST26* mutant (A) or of a *CST26* mutant transformed with GFP-tagged *CST26* (B). The profile for the transformed cells is similar to the wild-type profile (not shown), indicating that the level of m/z value 863 is restored and the relative ratio of m/z values 807 and 835 is also restored.
(0.05 MB PDF)
