# Supplementary material for: Characterization of Substrate Preference for Slc1p and Cst26p in Saccharomyces cerevisiae Using Lipidomic Approaches and an LPAAT Activity Assay
Source: PLoS One. 2010 Aug 4;5(8):e11956. doi: 10.1371/journal.pone.0011956 (PMC2915916; doi:10.1371/journal.pone.0011956)
Supplement: Table S1 — MRM transitions of individual phospholipids. (0.08 MB PDF) [file pone.0011956.s001.pdf]

Table S1

PG	[M-H]$^{-1}$→FA	PE	[M-H]$^{-1}$→FA	PC	[M+Cl]$^{-1}$→FA	PC	[M-H]$^{-1}$→FA	PI	[M-H]$^{-1}$→FA	PS	[M-H]$^{-1}$→FA	PA	[M-H]$^{-1}$→FA
PG-18:0/18:1	775.6→283.2	PE-18:0/18:1	744.6→283.2	PC-18:0/18:1	822.6→283.2	PC-18:1/18:0	772.5→281.2	PI-18:0/16:1	835.6→283.2	PS-18:0/16:1	760.6→283.2	PA-18:0/16:1	673.6→283.2
PG-18:0/16:0	749.6→283.2	PE-18:0/16:0	718.6→283.2	PC-18:0/16:0	796.6→283.2	PC-18:0/16:0	746.6→283.2	PI-18:0/16:0	837.6→283.2	PS-18:0/16:0	762.6→283.2	PA-18:0/16:0	675.6→283.2
PG-18:0/16:1	747.6→283.2	PE-18:0/16:1	716.6→283.2	PC-18:0/16:1	794.6→283.2	PC-18:0/16:1	744.6→283.2	PI-18:0/16:1	835.6→283.2	PS-18:0/16:1	760.6→283.2	PA-18:0/16:1	673.6→283.2
PG-18:0/14:0	721.6→283.2	PE-18:0/14:0	690.5→283.2	PC-18:0/14:0	768.5→283.2	PC-18:0/14:0	718.5→283.2	PI-18:0/14:0	809.6→283.2	PS-18:0/14:0	734.6→283.2	PA-18:0/14:0	647.6→283.2
PG-18:0/14:1	719.6→283.2	PE-18:0/14:1	688.5→283.2	PC-18:0/14:1	766.5→283.2	PC-18:0/14:1	716.5→283.2	PI-18:0/14:1	807.6→283.2	PS-18:0/14:1	732.6→283.2	PA-18:0/14:1	645.6→283.2
PG-18:0/12:0	693.5→283.2	PE-18:0/12:0	662.5→283.2	PC-18:0/12:0	740.5→283.2	PC-18:0/12:0	690.5→283.2	PI-18:0/12:0	781.6→283.2	PS-18:0/12:0	706.6→283.2	PA-18:0/12:0	619.6→283.2
PG-18:0/12:1	691.5→283.2	PE-18:0/12:1	660.5→283.2	PC-18:0/12:1	738.5→283.2	PC-18:0/12:1	688.5→283.2	PI-18:0/12:1	779.6→283.2	PS-18:0/12:1	704.6→283.2	PA-18:0/12:1	617.6→283.2
PG-18:0/10:0	665.5→281.1	PE-18:0/10:0	634.5→283.2	PC-18:0/10:0	712.5→283.2	PC-18:0/10:0	662.5→283.2	PI-18:0/10:0	753.6→283.2	PS-18:0/10:0	678.6→283.2	PA-18:0/10:0	591.6→283.2
PG-18:0/10:1	663.5→281.1	PE-18:0/10:1	632.5→283.2	PC-18:0/10:1	710.5→283.2	PC-18:0/10:1	660.5→283.2	PI-18:0/10:1	751.6→283.2	PS-18:0/10:1	676.6→283.2	PA-18:0/10:1	589.6→283.2
PG-18:0/8:0	637.5→281.1	PE-18:0/8:0	606.5→283.2	PC-18:0/8:0	684.5→283.2	PC-18:0/8:0	634.5→283.2	PI-18:0/8:0	725.6→283.2	PS-18:0/8:0	650.6→283.2	PA-18:0/8:0	563.6→283.2
PG-18:1/18:1	773.5→281.1	PE-18:1/18:0	744.5→281.2	PC-18:0/18:1	772.6→283.2	PC-18:1/18:0	772.5→281.2	PI-18:1/18:0	863.6→281.2	PS-18:1/18:0	788.6→281.2	PA-18:1/18:0	701.6→281.2
PG-18:1/16:0	747.5→281.1	PE-18:1/18:1	742.5→281.2	PC-18:1/18:1	820.5→281.2	PC-18:1/18:1	770.5→281.2	PI-18:1/18:1	861.6→281.2	PS-18:1/18:1	786.6→281.2	PA-18:1/18:1	699.6→281.2
PG-18:1/16:1	745.5→281.1	PE-18:1/16:0	716.5→281.2	PC-18:1/16:0	794.5→281.2	PC-18:1/16:0	744.5→281.2	PI-18:1/16:0	835.6→281.2	PS-18:1/16:0	760.6→281.2	PA-18:1/16:0	673.6→281.2
PG-18:1/14:0	719.6→281.1	PE-18:1/16:1	714.5→281.2	PC-18:1/16:1	792.5→281.2	PC-18:1/16:1	742.5→281.2	PI-18:1/16:1	833.6→281.2	PS-18:1/16:1	758.6→281.2	PA-18:1/16:1	671.6→281.2
PG-18:1/14:1	717.6→281.1	PE-18:1/14:0	688.5→281.2	PC-18:1/14:0	766.5→281.2	PC-18:1/14:0	716.5→281.2	PI-18:1/14:0	807.5→281.2	PS-18:1/14:0	732.5→281.2	PA-18:1/14:0	645.5→281.2
PG-18:1/12:0	691.6→281.1	PE-18:1/14:1	686.5→281.2	PC-18:1/14:1	764.5→281.2	PC-18:1/14:1	714.5→281.2	PI-18:1/14:1	805.5→281.2	PS-18:1/14:1	730.5→281.2	PA-18:1/14:1	643.5→281.2
PG-18:1/12:1	689.6→281.1	PE-18:1/12:0	660.5→281.2	PC-18:1/12:0	738.5→281.2	PC-18:1/12:0	688.5→281.2	PI-18:1/12:0	779.5→281.2	PS-18:1/12:0	704.5→281.2	PA-18:1/12:0	617.5→281.2
PG-18:1/10:0	663.6→281.1	PE-18:1/12:1	658.5→281.2	PC-18:1/12:1	736.5→281.2	PC-18:1/12:1	686.5→281.2	PI-18:1/12:1	777.5→281.2	PS-18:1/12:1	702.5→281.2	PA-18:1/12:1	615.5→281.2
PG-18:1/10:1	661.6→281.1	PE-18:1/10:0	632.5→281.2	PC-18:1/10:0	710.5→281.2	PC-18:1/10:0	660.5→281.2	PI-18:1/10:0	751.5→281.2	PS-18:1/10:0	676.5→281.2	PA-18:1/10:0	589.5→281.2
PG-18:1/8:0	635.6→281.1	PE-18:1/10:1	630.5→281.2	PC-18:1/10:1	708.5→281.2	PC-18:1/10:1	658.5→281.2	PI-18:1/10:1	749.5→281.2	PS-18:1/10:1	674.5→281.2	PA-18:1/10:1	587.5→281.2
PG-16:0/18:0	749.5→255.1	PE-18:1/8:0	604.5→281.2	PC-18:1/8:0	682.5→281.2	PC-18:1/8:0	632.5→281.2	PI-18:1/8:0	723.5→281.2	PS-18:1/8:0	648.5→281.2	PA-18:1/8:0	561.5→281.2
PG-16:0/18:1	747.5→255.1	PE-16:0/16:0	690.5→255.2	PC-16:0/18:1	794.5→255.2	PC-16:0/18:1	744.5→255.2	PI-16:0/18:1	835.6→255.1	PS-16:0/18:1	760.6→255.2	PA-16:0/18:1	673.6→255.2
PG-16:0/16:0	721.5→255.1	PE-16:0/18:1	716.5→255.2	PC-16:0/16:0	768.5→255.2	PC-16:0/16:0	718.5→255.2	PI-16:0/16:0	809.6→255.1	PS-16:0/16:0	734.6→255.2	PA-16:0/16:0	647.6→255.2
PG-16:0/16:1	719.5→255.1	PE-16:0/16:1	688.5→255.2	PC-16:0/16:1	766.5→255.2	PC-16:0/16:1	716.5→255.2	PI-16:0/16:1	807.6→255.1	PS-16:0/16:1	732.6→255.2	PA-16:0/16:1	645.6→255.2
PG-16:0/14:0	693.5→255.1	PE-16:0/14:0	662.5→255.2	PC-16:0/14:0	740.5→255.2	PC-16:0/14:0	690.5→255.2	PI-16:0/14:0	781.5→255.1	PS-16:0/14:0	706.5→255.2	PA-16:0/14:0	619.5→255.2
PG-16:0/14:1	691.5→255.1	PE-16:0/14:1	660.5→255.2	PC-16:0/14:1	738.5→255.2	PC-16:0/14:1	688.5→255.2	PI-16:0/14:1	779.5→255.1	PS-16:0/14:1	704.5→255.2	PA-16:0/14:1	617.5→255.2
PG-16:0/12:0	665.5→255.1	PE-16:0/12:0	634.5→255.2	PC-16:0/12:0	712.5→255.2	PC-16:0/12:0	662.5→255.2	PI-16:0/12:0	753.5→255.1	PS-16:0/12:0	678.5→255.2	PA-16:0/12:0	591.5→255.2
PG-16:0/12:1	663.5→255.1	PE-16:0/12:1	632.5→255.2	PC-16:0/12:1	710.5→255.2	PC-16:0/12:1	660.5→255.2	PI-16:0/12:1	751.5→255.1	PS-16:0/12:1	676.5→255.2	PA-16:0/12:1	589.5→255.2
PG-16:0/10:0	637.5→255.1	PE-16:0/10:0	606.5→255.2	PC-16:0/10:0	684.5→255.2	PC-16:0/10:0	634.5→255.2	PI-16:0/10:0	725.5→255.1	PS-16:0/10:0	650.5→255.2	PA-16:0/10:0	563.5→255.2
PG-16:0/10:1	635.5→255.1	PE-16:0/10:1	604.5→255.2	PC-16:0/10:1	682.5→255.2	PC-16:0/10:1	632.5→255.2	PI-16:0/10:1	723.5→255.1	PS-16:0/10:1	648.5→255.2	PA-16:0/10:1	561.5→255.2
PG-16:0/8:0	609.5→255.1	PE-16:0/8:0	578.5→255.2	PC-16:0/8:0	656.5→255.2	PC-16:0/8:0	606.5→255.2	PI-16:0/8:0	697.5→255.1	PS-16:0/8:0	622.5→255.2	PA-16:0/8:0	535.5→255.2
PG-16:1/18:0	747.5→253.1	PE-16:1/18:0	716.5→253.1	PC-16:1/18:0	794.5→253.1	PC-16:1/18:0	744.5→253.1	PI-16:1/18:0	835.5→253.1	PS-16:1/18:0	760.5→253.2	PA-16:1/18:0	673.5→253.2
PG-16:1/18:1	745.5→253.1	PE-16:1/18:1	714.5→253.1	PC-16:1/18:1	792.5→253.1	PC-16:1/18:1	742.5→253.1	PI-16:1/18:1	833.5→253.1	PS-16:1/18:1	758.5→253.2	PA-16:1/18:1	671.5→253.2
PG-16:1/16:0	719.5→253.1	PE-16:1/16:0	688.5→253.1	PC-16:1/16:0	766.5→253.1	PC-16:1/16:0	716.5→253.1	PI-16:1/16:0	807.5→253.1	PS-16:1/16:0	732.5→253.2	PA-16:1/16:0	645.5→253.2
PG-16:1/16:1	717.5→253.1	PE-16:1/16:1	686.5→253.1	PC-16:1/16:1	764.5→253.1	PC-16:1/16:1	714.5→253.1	PI-16:1/16:1	805.5→253.1	PS-16:1/16:1	730.5→253.2	PA-16:1/16:1	643.5→253.2
PG-16:1/14:0	691.5→253.1	PE-16:1/14:0	660.5→253.1	PC-16:1/14:0	738.5→253.1	PC-16:1/14:0	688.5→253.1	PI-16:1/14:0	779.5→253.1	PS-16:1/14:0	704.5→253.2	PA-16:1/14:0	617.5→253.2
PG-16:1/14:1	689.5→253.1	PE-16:1/14:1	658.5→253.1	PC-16:1/14:1	736.5→253.1	PC-16:1/14:1	686.5→253.1	PI-16:1/14:1	777.5→253.1	PS-16:1/14:1	702.5→253.2	PA-16:1/14:1	615.5→253.2
PG-16:1/12:0	663.5→253.1	PE-16:1/12:0	632.5→253.1	PC-16:1/12:0	710.5→253.1	PC-16:1/12:0	660.5→253.1	PI-16:1/12:0	751.5→253.1	PS-16:1/12:0	676.5→253.2	PA-16:1/12:0	589.5→253.2
PG-16:1/12:1	661.5→253.1	PE-16:1/12:1	630.5→253.1	PC-16:1/12:1	708.5→253.1	PC-16:1/12:1	658.5→253.1	PI-16:1/12:1	749.5→253.1	PS-16:1/12:1	674.5→253.2	PA-16:1/12:1	587.5→253.2
PG-16:1/10:0	635.5→253.1	PE-16:1/10:0	604.5→253.1	PC-16:1/10:0	682.5→253.1	PC-16:1/10:0	632.5→253.1	PI-16:1/10:0	723.5→253.1	PS-16:1/10:0	648.5→253.2	PA-16:1/10:0	561.5→253.2
PG-16:1/10:1	633.5→253.1	PE-16:1/10:1	602.5→253.1	PC-16:1/10:1	680.5→253.1	PC-16:1/10:1	630.5→253.1	PI-16:1/10:1	721.5→253.1	PS-16:1/10:1	646.5→253.2	PA-16:1/10:1	559.5→253.2
PG-16:1/8:0	607.5→253.1	PE-16:1/8:0	576.5→255.2	PC-16:1/8:0	654.5→255.2	PC-16:1/8:0	604.5→255.2	PI-16:1/8:0	695.5→253.1	PS-16:1/8:0	620.5→253.2	PA-16:1/8:0	533.5→253.2
PG-14:0/18:0	721.6→227.1	PE-14:0/18:1	688.5→227.1	PC-14:0/18:1	766.5→227.1	PC-14:0/18:1	716.5→227.1	PI-14:0/18:1	807.5→227.1	PS-14:0/18:1	732.5→227.1	PA-14:0/18:1	645.5→227.1
PG-14:0/18:1	719.6→227.1	PE-14:0/16:1	660.5→227.1	PC-14:0/16:1	738.5→227.1	PC-14:0/16:1	688.5→227.1	PI-14:0/16:0	781.5→227.1	PS-14:0/16:0	706.5→227.1	PA-14:0/16:0	619.5→227.1
PG-14:0/16:0	693.5→227.1	PE-14:0/14:0	634.5→227.1	PC-14:0/14:0	712.5→227.1	PC-14:0/14:0	662.5→227.1	PI-14:0/16:1	779.5→227.1	PS-14:0/16:1	704.5→227.1	PA-14:0/16:1	617.5→227.1
PG-14:0/16:1	691.5→227.1	PE-14:0/14:1	632.5→227.1	PC-14:0/14:1	710.5→227.1	PC-14:0/14:1	660.5→227.1	PI-14:0/14:0	753.5→227.1	PS-14:0/14:0	678.5→227.1	PA-14:0/14:0	591.5→227.1
PG-14:0/14:0	665.5→227.1	PE-14:0/12:0	606.5→227.1	PC-14:0/12:0	684.5→227.1	PC-14:0/12:0	634.5→227.1	PI-14:0/14:1	751.5→227.1	PS-14:0/14:1	676.5→227.1	PA-14:0/14:1	589.5→227.1
PG-14:0/14:1	663.5→227.1	PE-14:0/12:1	604.5→227.1	PC-14:0/12:1	682.5→227.1	PC-14:0/12:1	632.5→227.1	PI-14:0/12:0	725.5→227.1	PS-14:0/12:0	650.5→227.1	PA-14:0/12:0	563.5→227.1
PG-14:0/12:0	637.5→227.1	PE-14:0/10:0	578.5→227.1	PC-14:0/10:0	656.5→227.1	PC-14:0/10:0	606.5→227.1	PI-14:0/12:1	723.5→227.1	PS-14:0/12:1	648.5→227.1	PA-14:0/12:1	561.5→227.1
PG-14:0/12:1	635.5→227.1	PE-14:0/10:1	576.5→227.1	PC-14:0/10:1	654.5→227.1	PC-14:0/10:1	604.5→227.1	PI-14:0/10:0	697.5→227.1	PS-14:0/10:0	622.5→227.1	PA-14:0/10:0	535.5→227.1
PG-14:0/10:0	609.5→227.1	PE-14:0/8:0	550.5→227.1	PC-14:0/8:0	628.5→227.1	PC-14:0/8:0	578.5→227.1	PI-14:0/10:1	695.5→227.1	PS-14:0/10:1	620.5→227.1	PA-14:0/10:1	533.5→227.1
PG-14:0/10:1	607.5→227.1	PE-14:1/18:0	688.5→225.1	PC-14:1/18:0	766.5→225.1	PC-14:1/18:0	716.5→225.1	PI-14:0/8:0	669.5→227.1	PS-14:0/8:0	594.5→227.1	PA-14:0/8:0	507.5→227.1
PG-14:0/8:0	581.5→227.1	PE-14:1/18:1	686.5→225.1	PC-14:1/18:1	764.5→225.1	PC-14:1/18:1	714.5→225.1	PI-14:1/18:0	807.6→225.1	PS-14:1/18:0	732.6→225.1	PA-14:1/18:0	645.6→225.1
PG-12:0/12:0	609.5→199.1	PE-14:1/16:0	660.5→225.1	PC-14:1/16:0	738.5→225.1	PC-14:1/16:0	688.5→225.1	PI-14:1/18:1	805.6→225.1	PS-14:1/18:1	730.6→225.1	PA-14:1/18:1	643.6→225.1
PG-12:0/12:1	607.5→199.1	PE-14:1/16:1	658.5→225.1	PC-14:1/16:1	736.5→225.1	PC-14:1/16:1	686.5→225.1	PI-14:1/16:0	779.5→225.1	PS-14:1/16:0	704.5→225.1	PA-14:1/16:0	617.5→225.1
PG-12:0/10:0	581.5→199.1	PE-14:1/14:0	632.5→225.1	PC-14:1/14:0	710.5→225.1	PC-14:1/14:0	660.5→225.1	PI-14:1/16:1	777.5→225.1	PS-14:1/16:1	702.5→225.1	PA-14:1/16:1	615.5→225.1
PG-12:0/10:1	579.5→199.1	PE-14:1/14:1	630.5→225.1	PC-14:1/14:1	708.5→225.1	PC-14:1/14:1	658.5→225.1	PI-14:1/14:0	751.5→225.1	PS-14:1/14:0	676.5→225.1	PA-14:1/14:0	589.5→225.1
PG-12:0/8:0	553.5→143.1	PE-14:1/12:0	604.5→225.1	PC-14:1/12:0	682.5→225.1	PC-14:1/12:0	632.5→225.1	PI-14:1/14:1	749.5→225.1	PS-14:1/14:1	674.5→225.1	PA-14:1/14:1	587.5→225.1
PG-10:0/10:0	553.5→171.1	PE-14:1/12:1	602.5→225.1	PC-14:1/12:1	680.5→225.1	PC-14:1/12:1	630.5→225.1	PI-14:1/12:0	723.5→225.1	PS-14:1/12:0	648.5→225.1	PA-14:1/12:0	561.5→225.1
PG-10:0/10:1	551.5→169.1	PE-14:1/10:0	576.5→225.1	PC-14:1/10:0	654.5→225.1	PC-14:1/10:0	604.5→225.1	PI-14:1/12:1	721.5→225.1	PS-14:1/12:1	646.5→225.1	PA-14:1/12:1	559.5→225.1
PG-10:0/8:0	525.5→143	PE-14:1/10:1	574.5→225.1	PC-14:1/10:1	652.5→225.1	PC-14:1/10:1	602.5→225.1	PI-14:1/10:0	695.5→225.1	PS-14:1/10:0	620.5→225.1	PA-14:1/10:0	533.5→225.1
PG-8:0/8:0	497.5→143	PE-14:1/8:0	548.5→225.1	PC-14:1/8:0	626.5→225.1	PC-14:1/8:0	576.5→225.1	PI-14:1/10:1	693.5→225.1	PS-14:1/10:1	618.5→225.1	PA-14:1/10:1	531.5→225.1
		PE-12:0/12:0	578.5→199.1	PC-12:0/12:0	656.5→199.1	PC-12:0/12:0	606.5→199.1	PI-14:1/8:0	667.5→225.1	PS-14:1/8:0	592.5→225.1	PA-14:1/8:0	505.5→225.1
		PE-12:0/12:1	576.5→199.1	PC-12:0/12:1	654.5→199.1	PC-12:0/12:1	604.5→199.1	PI-12:0/12:0	697.5→199.1	PS-12:0/12:0	622.5→199.1	PA-12:0/12:0	535.5→199.1
		PE-12:0/10:0	550.5→199.1	PC-12:0/10:0	628.5→199.1	PC-12:0/10:0	578.5→199.1	PI-12:0/12:1	695.5→199.1	PS-12:0/12:1	620.5→199.1	PA-12:0/12:1	533.5→199.1
		PE-12:0/8:0	522.5→199.1	PC-12:0/8:0	600.5→199.1	PC-12:0/8:0	550.5→199.1	PI-12:0/10:0	669.5→199.1	PS-12:0/10:0	594.5→199.1	PA-12:0/10:0	507.5→199.1
		PE-12:1/12:0	576.5→197.1	PC-12:1/12:0	654.5→197.1	PC-12:1/12:0	604.5→197.1	PI-12:0/8:0	641.5→199.1	PS-12:0/8:0	566.5→199.1	PA-12:0/8:0	479.5→199.1
		PE-12:1/12:1	574.5→197.1	PC-12:1/12:1	652.5→197.1	PC-12:1/12:1	602.5→197.1	PI-12:1/12:0	695.5→197.1	PS-12:1/12:0	620.5→197.1	PA-12:1/12:0	533.5→197.1
		PE-12:1/10:0	548.5→197.1	PC-12:1/10:0	626.5→197.1	PC-12:1/10:0	576.5→197.1	PI-12:1/12:1	693.5→197.1	PS-12:1/12:1	618.5→197.1	PA-12:1/12:1	531.5→197.1
		PE-12:1/10:1	546.5→197.1	PC-12:1/10:1	624.5→197.1	PC-12:1/10:1	574.5→197.1	PI-12:1/10:0	667.5→197.1	PS-12:1/10:0	592.5→197.1	PA-12:1/10:0	505.5→197.1
		PE-12:1/8:0	520.5→197.1	PC-12:1/8:0	598.5→197.1	PC-12:1/8:0	548.5→197.1	PI-12:1/10:1	665.5→197.1	PS-12:1/10:1	590.5→197.1	PA-12:1/10:1	503.5→197.1
		PE-10:0/10:0	522.5→171.1	PC-10:0/10:0	600.5→171.1	PC-10:0/10:0	550.5→171.1	PI-12:1/8:0	639.5→197.1	PS-12:1/8:0	564.5→197.1	PA-12:1/8:0	477.5→197.1
		PE-10:0/8:0	496.5→171.1	PC-10:0/8:0	574.5→171.1	PC-10:0/8:0	524.5→171.1	PI-10:0/10:0	641.5→171.1	PS-10:0/10:0	566.5→171.1	PA-10:0/10:0	479.5→171.1
		PE-10:1/10:0	520.5→169.1	PC-10:1/10:0	598.5→169.1	PC-10:1/10:0	548.5→169.1	PI-10:0/8:0	613.5→171.1	PS-10:0/8:0	538.5→171.1	PA-10:0/8:0	451.5→171.1
		PE-10:1/8:0	494.5→169.1	PC-10:1/8:0	572.5→169.1	PC-10:1/8:0	522.5→169.1	PI-10:1/10:0	639.5→169.1	PS-10:1/10:0	564.5→169.1	PA-10:1/10:0	477.5→169.1
		PE-8:0/8:0	468.5→143	PC-8:0/8:0	546.5→143.1	PC-8:0/8:0	496.5→143.1	PI-10:1/8:0	611.5→169.1	PS-10:1/8:0	536.5→169.1	PA-10:1/8:0	449.5→169.1
								PI-8:0/8:0	585.5→143.0	PS-8:0/8:0	510.5→141	PA-8:0/8:0	423.5→141
