# Supplementary figures and images for: Characterization of Substrate Preference for Slc1p and Cst26p in Saccharomyces cerevisiae Using Lipidomic Approaches and an LPAAT Activity Assay
Source: PLoS One. 2010 Aug 4;5(8):e11956. doi: 10.1371/journal.pone.0011956 (PMC2915916; doi:10.1371/journal.pone.0011956)

Figure S1

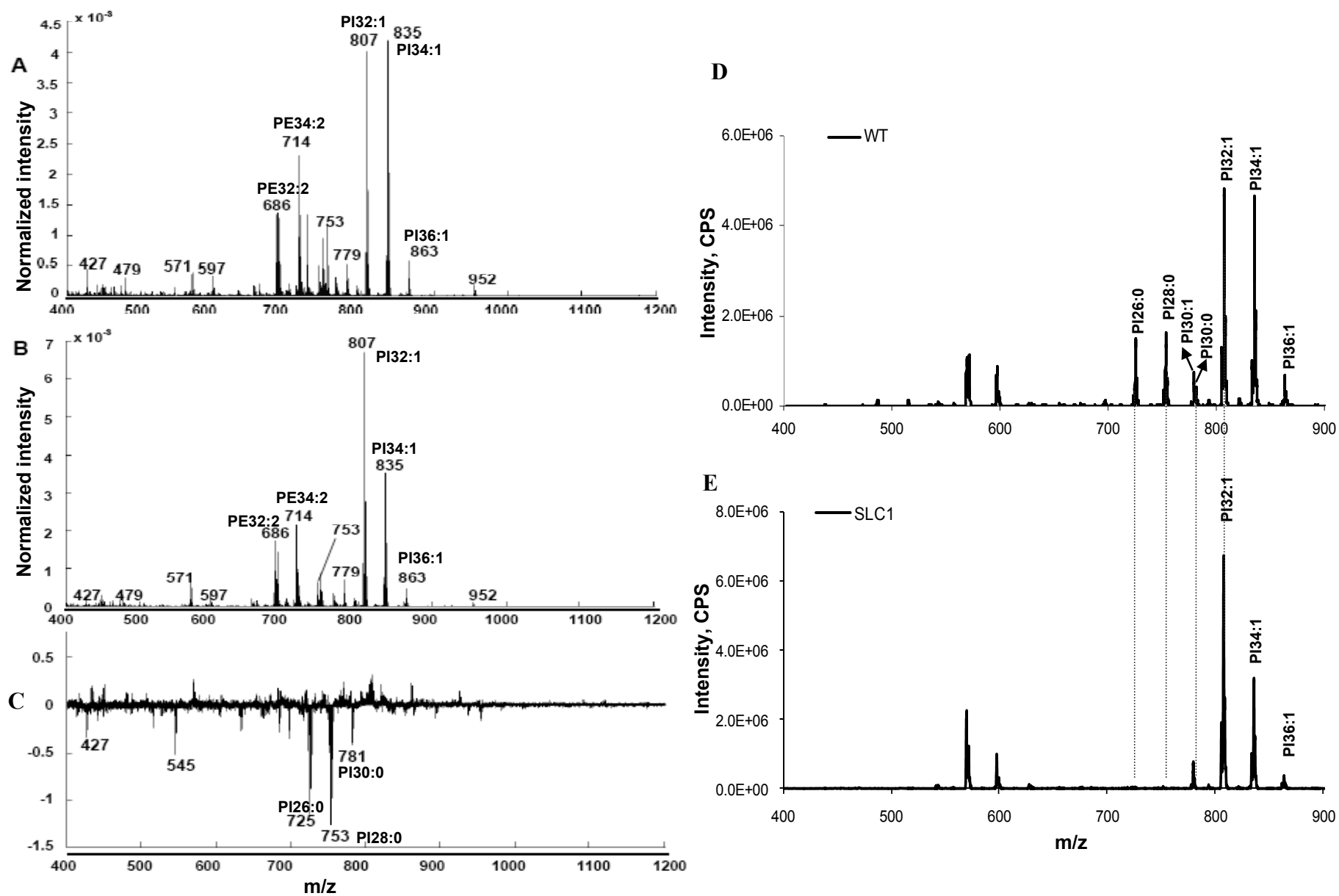

Supplement: Figure S1 — Unbiased lipid profiling of the SCL1 mutant. (A) Normalized lipid profile for the wild type; (B) normalized lipid profile for the SCL1 mutant; (C) differential plot of the SCL1 mutant compared to wild type; (D) and (e) precursor ion scan of ions at m/z 241 in wild type (WT) and ΔSLC1, respectively. (0.59 MB PDF) [file pone.0011956.s002.pdf]

Figure S2

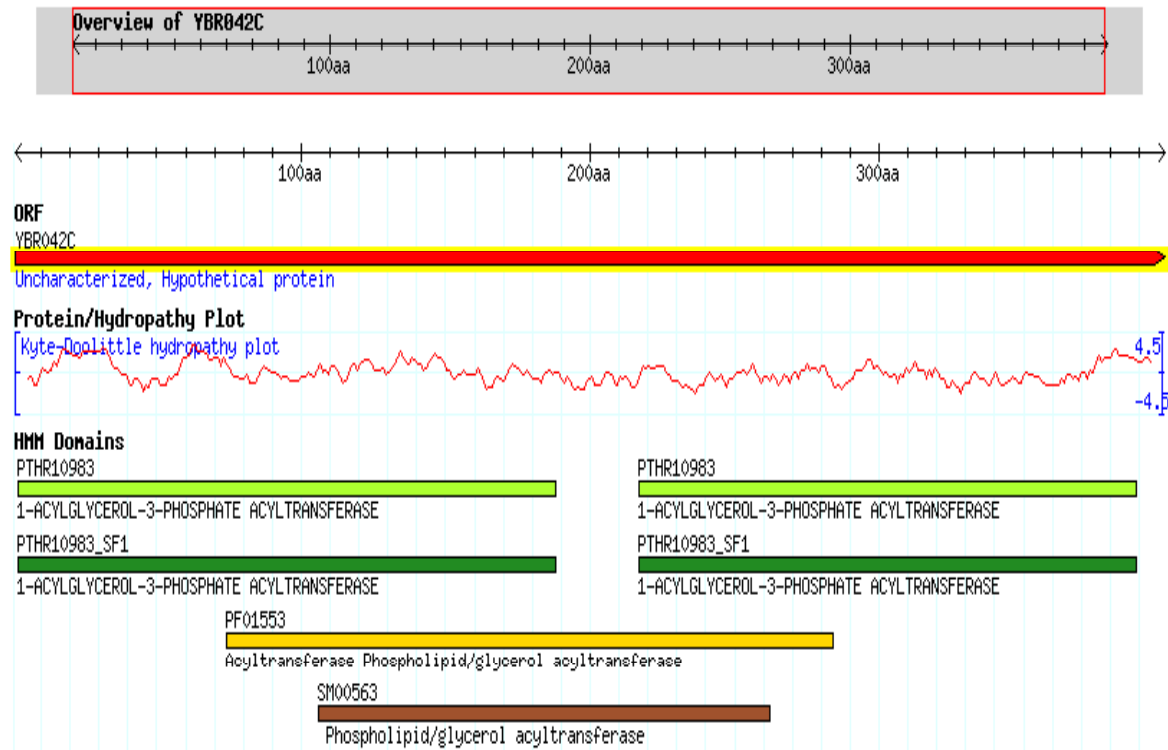

Supplement: Figure S2 — CST26 contains an acyltransferase domain. The domain information of YBR042C shows that it possesses a 1-acylglycerol-3-phosphate acyltransferase domain PTHR10983. (0.05 MB PDF) [file pone.0011956.s003.pdf]

Figure S3

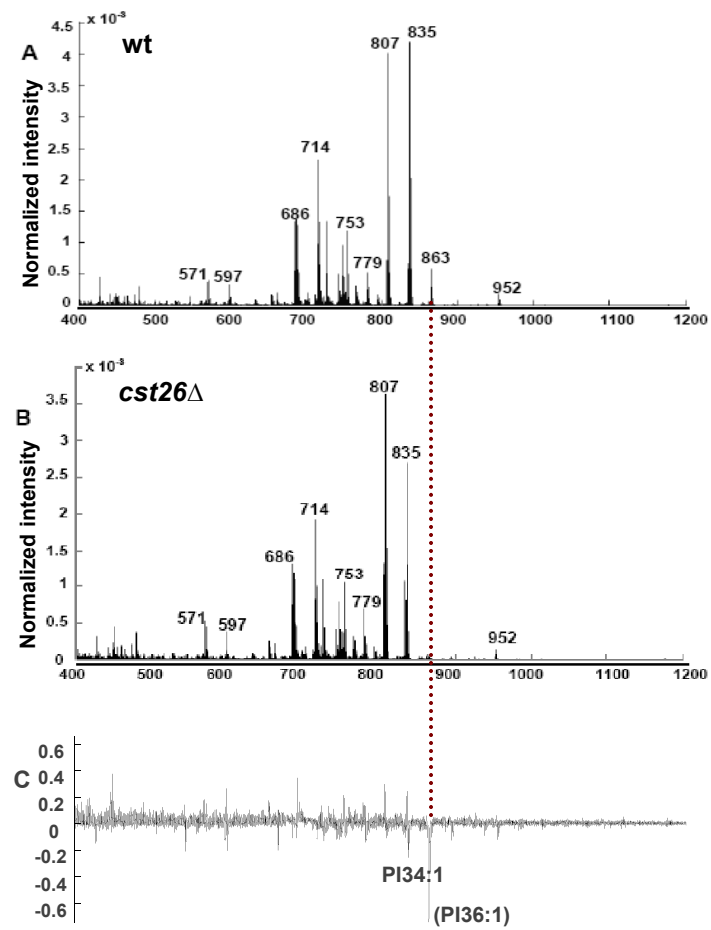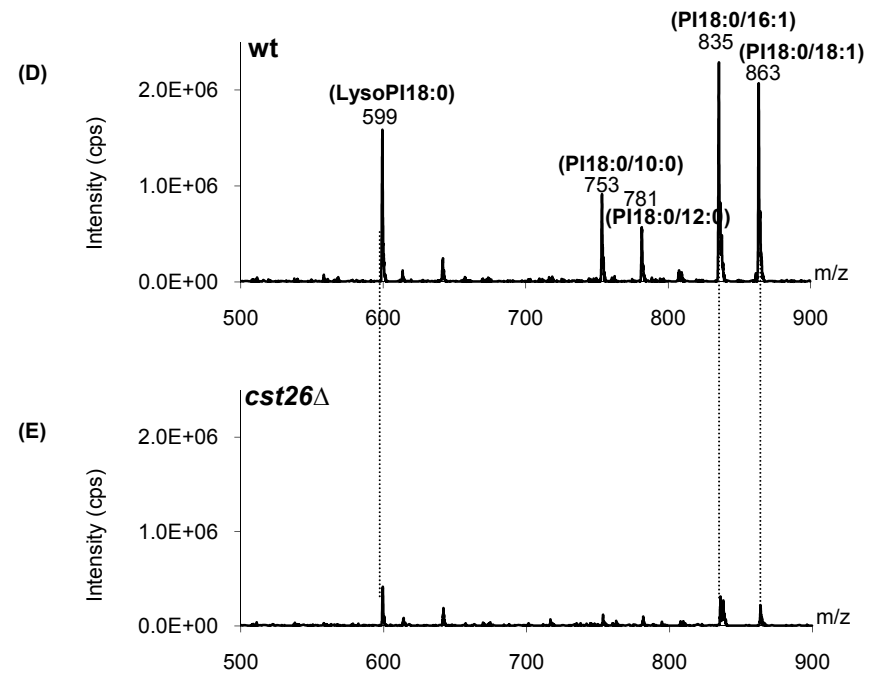

Supplement: Figure S3 — Unbiased lipid profiling of CST26 mutant. (A) Normalized lipid profile for the wild type; (B) normalized lipid profile for CST26 mutant; (C) differential plot of ΔCST26 compared to wild type; precursor ion scan of ions at m/z 283.1 (stearic acid) in wild-type (D) and cst26Δ cells (E). (0.31 MB PDF) [file pone.0011956.s004.pdf]

Figure S4

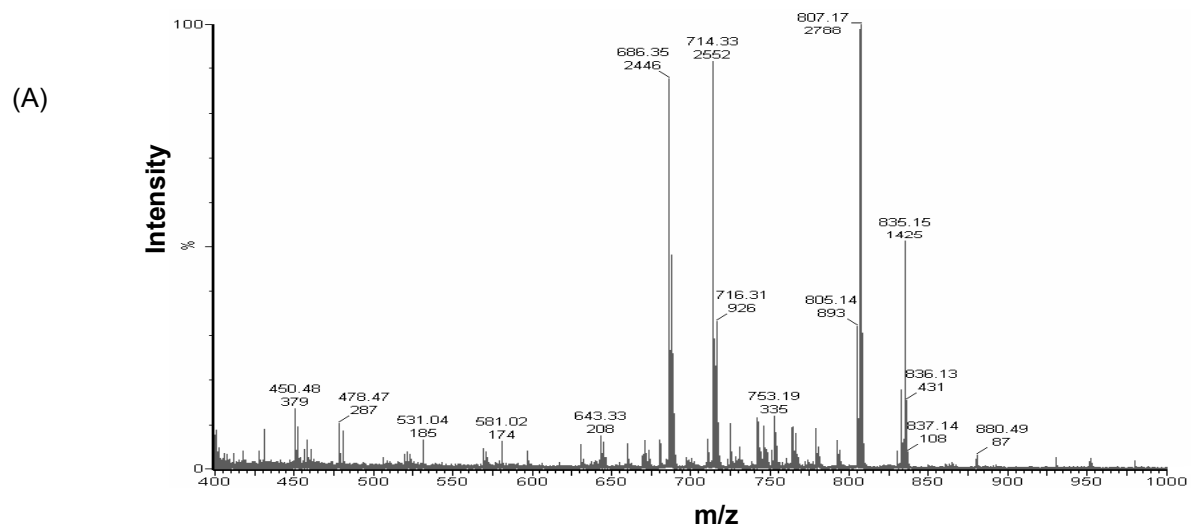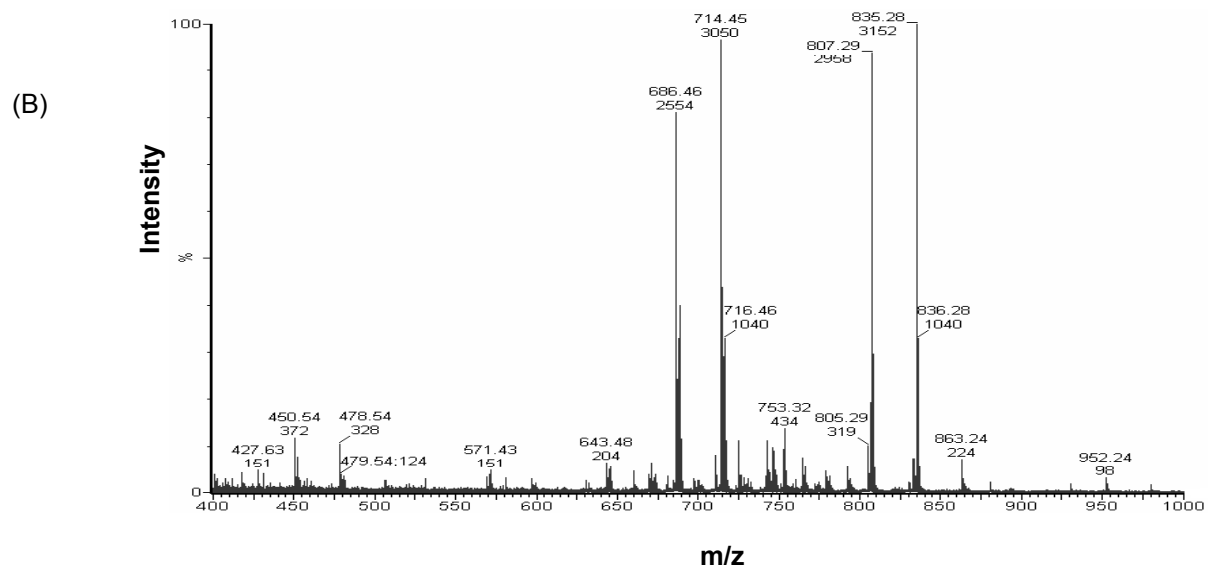

Supplement: Figure S4 — Mass spectrometric profile of a CST26 mutant (A) or of a CST26 mutant transformed with GFP-tagged CST26 (B). The profile for the transformed cells is similar to the wild-type profile (not shown), indicating that the level of m/z value 863 is restored and the relative ratio of m/z values 807 and 835 is also restored. (0.05 MB PDF) [file pone.0011956.s005.pdf]
